# Recent Evidence of the Role of CD4^+^ T Cell Subsets in IgG4-related Disease

**DOI:** 10.31662/jmaj.2024-0096

**Published:** 2024-12-06

**Authors:** Ryuta Kamekura, Hiroshi Sakamoto, Ryoto Yajima, Keisuke Yamamoto, Tsuyoshi Okuni, Motohisa Yamamoto, Hiroki Takahashi, Shingo Ichimiya, Kenichi Takano

**Affiliations:** 1 Department of Otolaryngology-Head and Neck Surgery, Sapporo Medical University School of Medicine, Sapporo, Japan; 2 Department of Human Immunology, Research Institute for Immunology, Sapporo Medical University School of Medicine, Sapporo, Japan; 3 Division of Rheumatology, Center for Antibody and Vaccine Therapy, Department of Rheumatology and Allergy, IMSUT Hospital, The Institute of Medical Science, The University of Tokyo, Tokyo, Japan; 4 Department of Rheumatology and Clinical Immunology, Sapporo Medical University School of Medicine, Sapporo, Japan

**Keywords:** IgG4-related disease, CD4^+^ T cell subsets, Tfh cells, Tph cells, Tfr cells, CD11c^+^CD21^-^ B cells, age-associated B cells

## Abstract

CD4^+^ T cells, the so-called T helper cells, are one of the main players in the human immune system, which can regulate acquired immunity. Dysfunction of the acquired immune system induces various chronic inflammatory diseases such as malignancies and autoimmune diseases. IgG4-related disease (IgG4-RD) is also a chronic inflammatory disease that is characterized by elevated serum IgG4 concentration and infiltration of IgG4-positive plasma cells in affected tissues. Despite that remarkable advances in understanding the pathogenesis of IgG4-RD have been on the rise, the detailed mechanisms by which IgG4-RD develops are still unknown. In fact, CD4^+^ T cells abundantly infiltrate at lesions of IgG4-RD, and they are also associated with the pathogenesis of other refractory chronic inflammatory diseases. Therefore, our focus was on CD4^+^ T cells, and we previously reported the roles of their subsets including regulatory T cells, CD4 cytotoxic T lymphocytes, T follicular helper (Tfh) cells, T follicular regulatory cells, and T peripheral helper (Tph) cells in IgG4-RD. Among the subsets, Tph cells play an important role in generating ectopic lymphoid structures at inflammatory sites. Moreover, we found that circulating Tph cells are increased in IgG4-RD patients. Unlike Tfh cells, Tph cells express high levels of chemokine receptors and cytotoxic molecules. Thus, they can infiltrate affected tissues and exert a cytotoxic function. Additionally, our latest observations demonstrated that Tph cells interact with extrafollicular B cells in affected tissues. Hence, Tph cells may collaborate with a specific B-cell subset, and they play a role in the maintenance of persistent fibroinflammation in lesions of IgG4-RD. Tph cells may have an important role to play in the pathogenesis of not only IgG4-RD but also other chronic inflammatory diseases. This review summarizes and discusses the possible pathologic roles of CD4^+^ T cell subsets including Tph cells in IgG4-RD.

## Introduction

**Figure 1.**
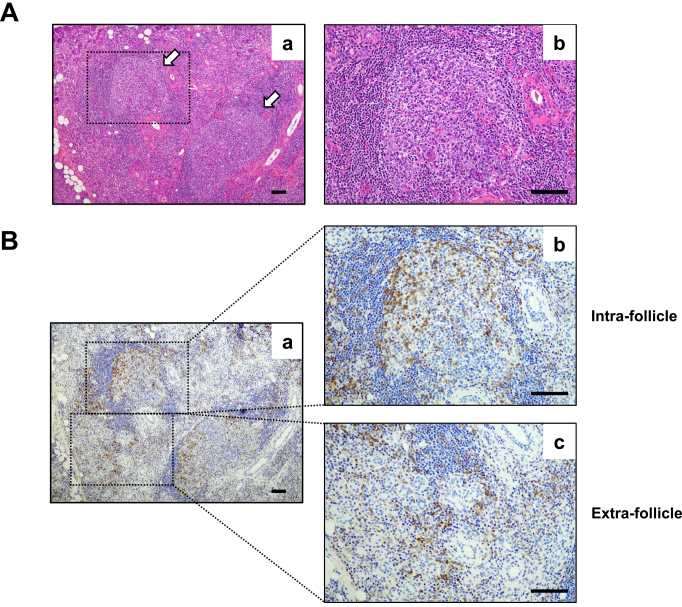
Histopathology of IgG4-related disease. A: Formation of ectopic lymphoid structures (arrows) in a submandibular gland from a patient with IgG4-related dacryoadenitis and sialoadenitis. (b) High-magnification image of the dotted square area in (a). A section of the submandibular gland was stained via hematoxylin and eosin. Scale bars are 100 μm. B: Immunohistochemistry staining for CD4 (brown). High-magnification images (b, intrafollicle; c, extrafollicle) of the dotted square areas in low-magnification image (a). Hematoxylin (blue) was utilized for nuclear staining. Scale bars are 100 μm.

IgG4-related disease (IgG4-RD) is an insidiously progressive fibroinflammatory disease that shows organ enlargement that mimics a tumor. It can affect the lacrimal glands, major salivary glands, pancreas, bile ducts, retroperitoneum, lungs, kidneys, aorta, and thyroid glands ^(1)^. It is characterized by chronic activation of the immune system and dysfunction of hormonal immunity. Nevertheless, the etiology of the disease is still unknown. Although reportedly, various immune cells related to innate and acquired immunity are involved in the immunological settings of IgG4-RD ^(2), (3)^, recent studies emphasized the central role of CD4^+^ T cells and B cells in the pathophysiology of IgG4-RD. Histopathologically, ectopic lymphoid structures (ELSs) that primarily comprise B cells and infiltration of CD4^+^ T cells around ELSs (intrafollicles and extrafollicles) are often found in affected tissues of IgG4-RD (Figure 1). Additionally, biologics including rituximab, which is an anti-CD20 antibody that depletes B cells, and abatacept, which is an anti-CTLA-4 antibody that inactivates T cells, have been shown to have good clinical responsiveness for IgG4-RD ^(4), (5), (6)^. Besides investigation of T helper (Th) cells and regulatory T (Treg) cells in the early period of IgG4-RD research, newly identified CD4^+^ T cell subsets, including T follicular helper (Tfh) cells, T follicular regulatory (Tfr) cells, CD4^+^ cytotoxic T lymphocytes (CD4^+^ CTLs), and T peripheral helper (Tph) cells, have been thoroughly explored. The results of studies strongly suggest the important roles of CD4^+^ T cells and B cells in the development of IgG4-RD. This review discusses the role of CD4^+^ T cell subsets in the pathogenesis of IgG4-RD described in recent reports and shows the results of our recent study on the interaction between Tph cells and extrafollicular B cells in IgG4-RD as a new topic of IgG4-RD pathophysiology.

## Treg Cells

IgG4-RD is histopathologically considered as infiltration of IgG4-positive plasma cells accompanied by organ enlargement with fibrosis in affected tissues ^(7), (8)^. Since interleukin (IL)-10 and transforming growth factor (TGF)-β are key cytokines for IgG4 class-switching and fibrosis, respectively ^(9), (10), (11)^, Treg cells have been focused on from the early period of IgG4-RD research as a pathognomonic source of IL-10 and TGF-β. Indeed, several studies have reported an increased number of Treg cells and an increased expression level of their master regulator, Foxp3, in both affected tissues and circulating lymphocytes in patients with IgG4-RD ^(12), (13), (14), (15), (16), (17)^. We also found increased numbers of Treg cells in the blood and affected tissues of patients with IgG4-RD (unpublished observation). Taken together, the results of these studies propose that Treg cells are involved in the disease pathogenesis in lesions of IgG4-RD; however, direct evidence of the function of Treg cells in IgG4-RD was not shown in these reports. To elucidate IgG4 class-switching and fibrosis caused by Treg cells in IgG4-RD, further studies are warranted.

## CD4^+^ CTLs

CD4^+^ T cells with cytotoxic activity (named CD4^+^ CTLs) have been observed in various immunological conditions including virus infection, autoimmune diseases, and malignancies ^(18), (19), (20)^ as well as after vaccination ^(21), (22)^. In general, CD4^+^ CTLs have been defined by the upregulation of surface molecules including CX3C-chemokine receptor 1 (CX3CR1), G protein-coupled receptor 56 (GPR56), and CD244 and are characterized by their unique function of secreting perforin, granzyme, and IFN-γ for MHC class II-expressing killing targets ^(18), (19), (23)^. Recent evidence proposes the involvement of CD4^+^ CTLs in IgG4-RD. The clonal expansion of CD4^+^ CTLs in inflamed tissue sites of IgG4-RD was first reported by Mattoo et al. These cells expressed SLAMF7, granzyme A (GZMA), IL-1β, and TGF-β, suggesting their capacity related to tissue inflammation and fibrosis. In fact, reportedly, apoptotic cells accumulate in tissues affected by IgG4-RD and show remarkable upregulation of HLA-DR ^(24)^. Interestingly, clinical remission induced by B-cell depletion with rituximab (which targets CD20) reduces the numbers of CD4^+^ CTLs in blood and affected organs in IgG4-RD ^(24), (25)^. These results reveal that activated B cells that migrate to inflamed tissues present an antigen to activated CD4^+^ T cells, which leads to the expansion of the T cells and their differentiation into CD4^+^ CTLs ^(1)^. Nonetheless, besides the lack of evidence in terms of the role of CD4^+^ CTLs in the pathogenesis of IgG4-RD, no study on the function of CD4^+^ CTLs in IgG4-RD has been conducted. To obtain direct evidence of cytotoxicity and fibrosis in affected tissues of IgG4-RD by these cells, additional studies are required in the future.

## Tfh Cells

Usually, abnormal infiltration of IgG4-positive plasma cells is observed in affected tissues of IgG4-RD ^(7)^. This depicts that dysregulation of the IgG4 class-switching underlies the pathogenesis of IgG4-RD. The ability of B cells to secrete class-switched high-affinity antibodies and to differentiate into plasma cells and memory B cells is dependent on signals from Tfh cells within germinal centers (GCs) ^(26), (27), (28)^. Tfh cells are a specialized class of effector helper CD4^+^ T cells and can secrete IL-4 and IL-10, which are key cytokines for IgG4 class-switching ^(9)^. Hence, Tfh cells have been considered to be one of the key players in the development of IgG4-RD. Tfh cells not only localize in lymphoid tissues including lymph nodes, spleens, and tonsils but also exist in blood circulation and lesional sites of extra-lymphoid tissues ^(27), (28), (29)^. Owing to the accessibility of blood samples, accumulating evidence has demonstrated the role of circulating Tfh (cTfh) cells in IgG4-RD. cTfh cells have been categorized into three subsets, namely, Tfh1, Tfh2, and Tfh17 cells, based on the expression of specific chemokine receptors, including CXC-chemokine receptor 3 (CXCR3) and CC-chemokine receptor 6 (CCR6). Tfh1, Tfh2, and Tfh17 cells can secrete restricted repertories of the cytokines IFN-γ, IL-4, and IL-17, respectively, as observed in conventional T helper cell subsets such as Th1, Th2, and Th17 cells ^(30), (31), (32)^. Among the subsets, cTfh2 cells and activated cTfh2 cells with a high expression level of programmed cell death 1 (PD-1, i.e., PD-1^hi^ cTfh2 cells) have been consistently associated with IgG4-RD ^(33), (34), (35)^. The number of cTfh2 cells correlates with clinical parameters such as the number of organs involved, circulating plasmablast numbers, and serum levels of IgG4 and IL-4 ^(33)^. Additionally, cTfh2 cells, but not cTfh1 and cTfh17 cells, were reported to induce IgG4 class-switching in B cells in *in vitro* experiments ^(34)^. These findings suggested that cTfh2 cells in patients with IgG4-RD embody Tfh cells in affected tissues of IgG4-RD that are involved in plasmablast differentiation and IgG4 class-switching. As recently reported by Higashioka et al., activated SLAMF7^+^ cTfh1 cells can highly produce IL-10 and have a pathologic role to play in IgG4 production along with Tfh2 cells in IgG4-RD ^(36)^. Moreover, activated cTfh1 cells were also shown to be increased in IgG4-RD and to be correlated with disease activity but not with serum IgG4 levels ^(34)^. Besides expanded Tfh cells in the blood, there has been accumulating evidence of the accumulation of Tfh cells in tissues affected by IgG4-RD. Our group and others have shown abundant infiltration of Tfh cells in affected submandibular glands of patients with IgG4-RD ^(17), (29), (35)^. We further revealed that lesional Tfh cells isolated from submandibular glands of patients with IgG4-related dacryoadenitis and sialoadenitis (IgG4-DS) demonstrated high expression levels of B-cell lymphoma 6 (Bcl6), CXC-chemokine ligand 13 (CXCL13), IL-10, and activation markers such as PD-1 and inducible T cell co-stimulator and had a greater capacity than tonsillar Tfh cells to help B cells produce IgG4 ^(29)^. Additionally, Maehara et al. revealed that the expansion of IL-4^+^BATF^+^ Tfh cells in both secondary and tertiary lymphoid organs from patients with IgG4-RD is associated with IgG4 class-switching ^(37)^. More recently, single-cell RNA sequencing analysis showed that IL-10^+^LAG3^+^ Tfh cells infiltrate the affected submandibular glands of IgG4-RD patients ^(38)^. Taken together, the results indicate that activated Tfh cells abundantly infiltrate affected tissues of IgG4-RD and have a pivotal role to play in the pathogenesis of IgG4-RD. Fundamental questions that are yet to be answered revolve around whether cTfh2 cells and resident Tfh cells in affected tissues of patients with IgG4-RD have the same origin and, if so, how cTfh cells migrate from or into the affected tissues. Our observations demonstrated that Tfh cells in affected tissues could not be categorized into the three subsets (Tfh1, Tfh2, and Tfh17 cells) because of the loss of expression of CXCR3 and CCR6 (unpublished observations). To address these questions, further studies on Tfh cells in IgG4-RD are required.

## Tfr Cells

Tfr cells have been characterized as a unique subset of CD4^+^ T cells that originate from natural regulatory T cells and participate in the regulation of GC formation and class switch recombination (CSR) of B cells in collaboration with Tfh cells ^(39), (40), (41)^. They express CXC-chemokine receptor 5 (CXCR5), which is also shared by B cells and Tfh cells. They, like Tfh cells, are dependent on the transcriptional repressor protein Bcl6 for their development; however, unlike Tfh cells, they express the canonical Treg master regulatory transcription factor Foxp3 ^(39), (40)^. To exert GC responses, they produce IL-10 and TGF-β for the direct regulation of B cells and Tfh cells. Although they are widely considered inhibitors of GC reactions ^(39), (40)^, Laidlaw et al. and our group have presented evidence that they promote GC through the production of IL-10 ^(41), (42)^. Miles et al. reported that Tfr cells proportionately and numerically proliferate during human immunodeficiency virus (HIV) infection and contribute to inefficient GC responses and then inhibit HIV clearance ^(43)^. Other studies have revealed a decreased number of circulating Tfr (cTfr) cells in various autoimmune diseases ^(44), (45), (46), (47)^. By contrast, the pathological significance of Tfr cells in IgG4-RD had not been explored. Thus, to address the question of whether Tfr cells are associated with the pathogenesis of IgG4-RD, we examined Tfr cells. Our studies showed that the number of Tfr cells was increased in the blood and inflamed submandibular glands from patients with IgG4-DS. The percentage of Tfr cells was positively correlated with clinical parameters including serum level of IgG4 and number of involved organs in IgG4-RD patients. Interestingly, the number of IL-10-producing circulating Tfr cells in patients with IgG4-RD was increased when compared with that in healthy older adults, which suggests the involvement of Tfr cells in IgG4-specific CSR in lesions of IgG4-RD ^(42)^. Recently, single-cell transcriptomics showed that abundant Tfr cells with suppressor-associated features infiltrated affected tissues from patients with IgG4-RD ^(48)^, which supported our results. Collectively, these findings provide a novel insight into the role of Tfr cells in the pathogenesis of IgG4-RD.

## Tph Cells

In 2017, Rao et al. revealed in their study on rheumatoid arthritis (RA) an unidentified subset of CD4^+^ T cells known as Tph cells (PD-1^hi^CXCR5^-^CD4^+^ cells) ^(49)^. Tph cells accumulate in the inflamed synovium of RA and exhibit Tfh cell-like features to produce factors linked to B-cell help, including IL-21 and CXCL13, which is a ligand of CXCR5. Hence, Tph cells are considered to have the capacity to recruit B cells and Tfh cells, which leads to the formation of ELSs (Figure 1), in nonlymphoid tissues. Unlike Tfh cells, Tph cells do not express high levels of Bcl6 and instead demonstrate elevated levels of Blimp1, which opposes the actions of Bcl6 as a counter-regulator, and Sox4 ^(50), (51)^. Tph cells also have a high expression profile of chemokine receptors, including CCR2, CCR5, and CX3CR1 (a fractalkine receptor) that induce their migration to inflamed sites ^(49)^. Therefore, Tph cells are distinguished from Tfh cells in their surface phenotypes, migratory capacity, and transcriptional regulation ^(49)^. Several recent studies on Tph cells have reported their pathological roles in various autoimmune diseases including systemic lupus erythematosus (SLE) ^(52), (53), (54)^, primary Sjögren’s syndrome ^(55)^, systemic sclerosis ^(56)^, type 1 diabetes ^(57)^, and inflammatory bowel diseases ^(58)^ as well as RA ^(49), (59)^, and in malignant tumors ^(60)^. In those studies, it was consistently shown that expansion of Tph cells is associated with autoimmune diseases ^(49), (52), (53), (54), (55), (56), (57), (59)^. Additionally, Gu-Trantien et al. reported that CD4^+^ T cells with a Tph cell-like phenotype were found in breast cancer tissues and that they have a possible regulatory function in immune responses against tumor cells ^(60)^. We first reported a possible pathological role of Tph cells in IgG4-RD ^(61)^. Our results have shown that circulating Tph (cTph) cells were significantly increased within CD4^+^ T cells in patients with IgG4-RD when compared with those in healthy volunteers. Moreover, we found that their percentage was positively correlated with clinical parameters such as serum levels of IgG4, soluble interleukin-2 receptor, and number of involved organs in IgG4-RD patients. Furthermore, we found that such Tph cells frequently expressed GZMA, which is related to a cytotoxic property. Glucocorticoid treatment led to a numerical reduction of Tph cells in parallel with the reduction of serum IgG4 level ^(61)^. Caielli et al. also reported that Tph cells (CXCR5^-^CXCR3^+^PD-1^hi^CD4^+^ cells) upregulated transcription factors associated with cytotoxic T lymphocytes including *GZMA*, *GAMB*, *PRF1*,* ZNF683*, *RUNX3*,* EOMES*, and *TBX21* ^(62)^. More recently, we reported the functional significance of CX3CR1^+^ Tph cells in IgG4-RD ^(63)^. CX3CR1 is a chemokine receptor of fractalkine (CX3CL1), which is a unique ligand for the coordination of inflammation as well as the wound healing process ^(64)^. The expression of CX3CR1 was demonstrated to be strongly associated with the cytotoxic potential of CD8^+^ T cells in both humans and mice ^(65)^. This ligand-receptor pair has been proposed to contribute to inflammatory diseases including autoimmune diseases ^(66), (67)^ and allergic airway diseases ^(68)^. The results of those studies revealed that cTph cells from patients with IgG4-RD highly expressed CX3CR1. Percentages of CX3CR1^+^ cTph cells were significantly correlated with clinical parameters including IgG4-RD Responder Index, number of involved organs, and serum level of soluble IL-2 receptor. CX3CR1^+^ Tph cells abundantly possessed cytotoxic T lymphocyte-related molecules such as GZMA, perforin, and G protein-coupled receptor 56. Functional assays revealed their cytotoxic potential against vascular endothelial cells and ductal epithelial cells ^(63)^. Our findings strongly suggest that circulating Tph cells have a pivotal role to play in the pathogenesis of IgG4-RD. A recent topic of Tph cells is that Tph2 (CXCR3^−^CCR6^−^ Tph) cells, which are one of the Tph cell subsets, have cytotoxic properties and are increased in peripheral blood of patients with IgG4-RD ^(69)^. Nevertheless, the role of Tph2 cells in IgG4-RD pathogenesis is still unknown. Hence, the functional role of Tph2 cells as well as the functional roles of other Tph subsets (Tph1 and Tph17 cells) must be elucidated in future experiments.

## Interaction between Tph Cells and Extrafollicular B Cells in IgG4-RD

**Figure 2.**
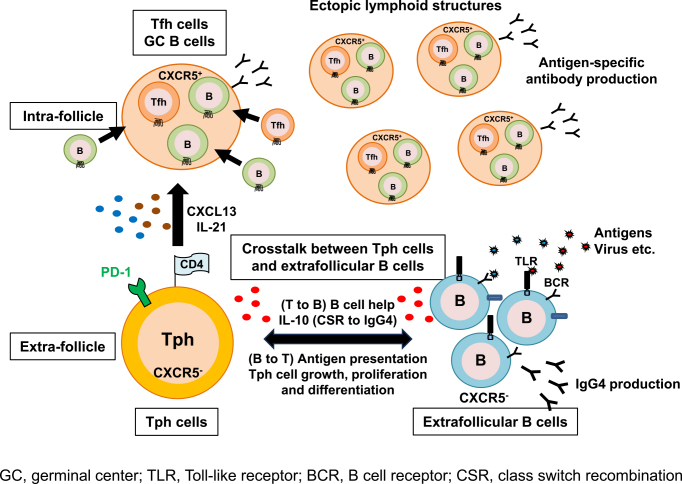
A model for the functional roles of PD-1^hi^CXCR5^−^ T peripheral helper (Tph) cells and extrafollicular B cells in affected organs of IgG4-RD. Tph cells accumulate in the inflamed tissues and produce factors associated with B-cell help, including IL-21 and CXCL13, which is a ligand of CXCR5. Therefore, it is believed that Tph cells can recruit B cells and Tfh cells, leading to the formation of ectopic lymphoid structures in nonlymphoid tissues. Tph cells lack CXCR5 expression and express a lower level of Bcl-6. Therefore, they are localized outside ELSs and exert their functions as B cell helpers. Tph cells may interact with extrafollicular follicular B cells (CD11c^+^CD21^−^ cells, referred to as age-associated B cells, ABCs) that also lack CXCR5 expression. From our recent observations, Tph cells interact with extrafollicular B cells in affected tissues of IgG4-RD. Additionally, CD11c^+^CD21^−^ B cells in IgG4-RD are a main producer of IgG4. Tph cells interact with CD11c^+^CD21^−^ B cells in extrafollicles, and IL-10 produced by Tph cells may induce CSR to IgG4 in CD11c^+^CD21^−^ B cells. Conversely, CD11c^+^CD21^−^ B cells may influence cell growth, proliferation, and differentiation of Tph cells as antigen-presenting cells.

Unlike Tfh cells, Tph cells lack CXCR5 expression and express a lower level of Bcl-6. Thus, Tph cells are localized outside ELSs and exert their functions as B-cell helpers ^(70)^. Tph cells are thought to interact with extrafollicular “atypical memory” CD11c^+^CD21^−^ B cells (referred to as age-associated B cells, ABCs) that also lack CXCR5 expression ^(71), (72), (73)^. Wang et al. revealed that CD11c^+^CD21^−^CXCR5^−^ B cells were highly expanded in patients with active SLE ^(74)^. Additionally, the frequency of ABCs correlated with that of CXCR3^+^PD-1^hi^CD4^+^ T (Tph) cells ^(62)^. In support of this observation, we investigated the frequency of CD11c^+^CD21^−^ B cells in peripheral blood from patients with IgG4-RD. Expectedly, the percentages of circulating Tph cells and CD11c^+^CD21^−^ B cells in IgG4-RD patients were significantly larger than those in healthy volunteers. Further analysis demonstrated that there was a marked positive correlation between the percentage of not Tfh cells but Tph cells with the percentage of CD11c^+^CD21^−^ B cells (unpublished observation). These findings suggested that Tph cells interact with extrafollicular B cells in affected tissues of IgG4-RD. Interestingly, our latest observations showed that there was a larger population of IgG4-positive cells in CD11c^+^CD21^−^ B cells than in CD11c^−^CD21^+^ B cells in IgG4-RD (unpublished observation), which suggests that CD11c^+^CD21^−^ B cells in IgG4-RD are a main producer of IgG4. Tph cells produce a larger amount of IL-10 when compared with Tfh cells and provide B-cell help through IL-10 in SLE ^(62)^. Considering that IL-10 is a key cytokine for CSR to IgG4 ^(9), (11)^ and Tph cells interact with CD11c^+^CD21^−^ B cells in extra follicles, IL-10 produced by Tph cells may induce CSR to IgG4 in CD11c^+^CD21^−^ B cells. Conversely, CD11c^+^CD21^−^ B cells may influence cell growth, proliferation, and differentiation of Tph cells as antigen-presenting cells. The mechanisms by which Tph cells help CD11c^+^CD21^−^ B cells to produce IgG4 and by which CD11c^+^CD21^−^ B cells control the functions of Tph cells are yet to be clarified. To elucidate the mechanisms, further *in vitro* functional analysis of the relationship between Tph cells and CD11c^+^CD21^−^ B cells is warranted in a future study. Taken together, the findings indicate that Tph cells might have a bidirectional relationship with CD11c^−^CD21^+^ B cells and play a pivotal role in the pathogenesis of IgG4-RD (Figure 2). Elucidating their relationship may lead to a deeper understanding of the pathogenesis of persistent inflammation in not only IgG4-RD but also other autoimmune diseases including SLE and RA.

## Conclusion

Recent advances in our knowledge of immunological mechanisms by which IgG4-RD develops are remarkable. One example is the appearance of newly identified CD4^+^ T cell subsets such as Tph cells and Tfh cells as key players in IgG4-RD pathogenesis. Additionally, the crosstalk between Tph cells and CD11c^+^CD21^−^ B cells, which is mentioned in this review, might be a novel mechanism by which IgG4-RD develops and those cells are promising therapeutic targets. Although treatment with glucocorticoids is still effective for IgG4-RD, it often has adverse effects and relapse frequently takes place after tapering or discontinuing administration of glucocorticoids ^(75)^. A new strategy for overcoming such problems for IgG4-RD must be developed. Safe glucocorticoid-sparing treatments would be beneficial for such patients. Hence, future studies must focus on discovering new therapeutic targets from basic research and development of new therapeutic agents such as biologics for IgG4-RD. Additionally, experimental results and knowledge regarding the pathogenesis of IgG4-RD from direct examination of inflamed tissues from patients with IgG4-RD must be considered. Although the availability of clinical specimens is limited, studies that use affected tissues and blood from IgG4-RD patients must be carried out.

## Article Information

This article is based on the study, which received the Medical Research Encouragement Prize of The Japan Medical Association in 2023.

### Conflicts of Interest

None

### Sources of Funding

This work was supported by grants-in-aid for scientific research from the Japanese Society for the Promotion of Science including #22K09670 (K.Y.) and #24K12651 (T.O.) and from the Takeda Science Foundation (R.K.).

### Acknowledgement

We thank our colleagues for their constructive discussion and technical support.
